# The Superior Ability of Human BDCA3^+^ (CD141^+^) Dendritic Cells (DCs) to Cross-Present Antigens Derived From Necrotic Lung Cancer Cells

**DOI:** 10.3389/fimmu.2020.01267

**Published:** 2020-06-19

**Authors:** Fei-fei Gu, Kai Zhang, Li-li Ma, Yang-yang Liu, Chang Li, Yue Hu, Qi-fan Yang, Jin-yan Liang, Yu-lan Zeng, Yan Wang, Li Liu

**Affiliations:** ^1^Cancer Center, Union Hospital, Tongji Medical College, Huazhong University of Science and Technology, Wuhan, China; ^2^Department of Oncology, Wuhan Brain Hospital, Wuhan, China; ^3^Analysis and Testing Center, Institute of Hydrobiology, Chinese Academy of Sciences, Wuhan, China

**Keywords:** lung cancer, dendritic cell, antitumor vaccine, EGFR, cytotoxic T lymphocyte, TLR

## Abstract

Dendritic cells (DCs) play a key role in initiating and regulating the immune responses to pathogens, self-antigens, and cancers. Human blood DCs comprise a family of different subsets: plasmacytoid DCs (pDCs) and CD16^+^, CD1c/BDCA1^+^, and BDCA3^+^ (CD141^+^) myeloid DCs and possess different phenotypes and functional characteristics. Lung cancer is the most common cancer, with the highest morbidity and mortality in the world. However, which DC subset plays a leading role in the lung cancer immune responses is unclear. We reanalyzed C-type lectin domain family 9 member A (CLEC9A) and CD141 (THBD) gene expression profiles from the Cancer Genome Atlas (TCGA) database and performed the Kaplan-Meier survival analysis of overall survival for several cancers according to their expression levels. Next, we investigated the capacities of five human blood DC subsets to stimulate T cell proliferation and capture, process and (cross-) present tumor antigen. Human BDCA3^+^ (CD141^+^) DCs have a superior capacity to stimulate allogeneic CD4^+^T cells proliferation and induce superior Th1 response compared with other DC subsets. Interestingly, toll-like receptor (TLR) agonists have little effect on DCs to induce the proliferation of naïve CD4^+^ T cells, but contribute to their differentiation. Importantly, BDCA3^+^ (CD141^+^) DCs possess the most potent ability to cross-present human tumor antigen after their uptake of necrotic lung cancer cells despite their lower antigen uptake. These findings suggest that human BDCA3^+^ (CD141^+^) DCs are critical mediators of cytotoxic T lymphocyte responses against EGFR-positive lung cancer. Therefore, our findings may provide theoretical basis for the development of DC-based antitumor vaccines.

## Introduction

Lung cancer has recently become the leading cause of cancer-related death worldwide. In 2015, there were approximately 4.29 million new cancer patients and 2.81 million deaths in China, and the incidence and mortality of lung cancer were ranked first (1). Treatment strategies for lung cancer have evolved with an emphasis on immunotherapy since conventional therapeutic approaches, such as surgery, radiotherapy, chemotherapy, and targeted therapy, have hardly improved the outcomes of advanced lung cancer (2).

DCs are the most potent antigen presenting cells (APC) and critical regulators of immune responses. According to their origins, DCs can be divided into different subsets: myeloid BDCA3^+^ (CD141^+^) DCs, CD16^+^ DCs and CD1c^+^ DCs and plasmacytoid DCs (pDCs) (3–5). DCs ingest, process, and present antigens, thereby activating the initial T cells to exert anti-tumor activity (6). Although DC based therapeutic vaccines have been applied in the treatment of malignancies, their efficacy is unsatisfactory (7). As human blood DCs comprise only 1% of the total PBMC fraction (4), most clinical studies use DCs cultured *ex vivo*, starting from precursor cells as the basis of immunotherapy vaccines (8). The precursors commonly used to generate DC vaccines are monocytes and CD34^+^ hematopoietic progenitor cells (8, 9). Nonetheless, monocyte-derived DCs (MoDC) are the most commonly used DC type in DC vaccine formulations (10). This type of DC vaccine is usually generated by isolating CD14^+^ monocytes from PBMCs and culturing them *in vitro* for 6 days into immature DCs with GM-CSF and IL-4. The next step is to induce their maturation and load them with tumor antigens for another 2 days before use for clinical treatment (11). However, MoDC cannot represent the physiological function of human blood DCs. Then Karolina Palucka et al. suggested that the lack of subset specificity in the application of DC vaccines was an important reason for the poor outcome (12).

Antigens are processed into short peptides by DC after their uptake and then assembled with MHC molecules. Ultimately, peptide–MHC complexes are expressed on the cell membrane. The peptide–MHC complexes can bind only to the matched TCR of T cells and then stimulate T cells in the presence of costimulatory molecules. Peptide–MHC class I complexes activate CD8^+^ T cells, while peptide–MHC class II complexes activate CD4^+^ T cells (13). Cross-presentation is the process by which ingested exogenous antigens can gain access to the MHC class I processing pathway of DCs to elicit CD8^+^ CTL response (14). Cross-presentation provides a way for antigen-presenting cells to recognize exogenous antigens and is essential for the induction of protective CD8^+^ T cell immunity against tumor and pathogenic bacteria (15). Cross-presentation of acquired exogenous antigen to CD8^+^ CTLs is essential for initiating the anti-tumor immune responses (16). Epidermal growth factor receptors (EGFRs) are important targets of non-small-cell lung cancer (NSCLC) therapy. NSCLC accounts for 75–80% of total lung cancers, and more than 60% of NSCLC expresses EGFR (17). Moreover, EGFR_853−861_ is an immunogenic HLA-A^*^0201-restricted epidermal growth factor receptor-specific T-cell epitope (18). Thus, EGFR was chosen as a target antigen in this study.

CLEC9A is a C-type lectin-like receptor and acts as a sensor of necrotic cells and regulator of cross-priming. CLEC9A can mediate endocytosis, but not phagocytosis. Expression of human CLEC9A is highly restricted in peripheral blood, being detected only on BDCA3^+^ DCs and on a small subset of CD14^+^CD16^−^ monocytes (19). BDCA3^+^ (CD141^+^) DCs have been established as an important functionally distinct human DC subtype with characteristics similar to those of the mouse CD8^+^ DC subset (6). We speculated that BDCA3^+^ (CD141^+^) DCs play an important role in antitumor immune response.

Here, we evaluated the abilities of ingesting, processing and cross-presenting lung cancer-associated antigen as well as the capacity to activate T cells among the aforementioned four subtypes of DCs and monocyte-derived dendritic cells (MoDC) in human peripheral blood. For the first time, we proved that BDCA3^+^ (CD141^+^) DCs have the strongest capacity to activate allogeneic naïve CD4^+^ T cells and potently induces them to differentiate into Th1 cells. Importantly, BDCA3^+^ (CD141^+^) DCs have the strongest ability to cross-present soluble antigen peptides and necrotic lung cancer cell-associated antigens to specific CD8^+^ T cells. These findings will help us analyze the mechanisms underlying the immune responses elicited by DCs and their potential clinical relevance.

## Methods

### DC Separation and Culture

The study was reviewed and approved in 2013 by the Ethics Committee of Tongji Medical College, Huazhong University of Science and Technology. DCs were isolated from buffy coats obtained from healthy volunteers after written informed consent according to the Declaration of Helsinki and medical ethics. Peripheral blood mononuclear cells (PBMCs) were purified from buffy coats via Ficoll density gradient centrifugation (Haoyang, Tianjin, China). CD1c^+^ DCs, BDCA3^+^ (CD141^+^) DCs, CD16^+^ DCs, and pDCs were isolated from PBMCs with a CD1c^+^ DC isolation kit, CD141 (BDCA3) isolation kit, CD16^+^ monocyte isolation kit and antiBDCA-4–conjugated magnetic microbeads, respectively (all Miltenyi Biotec, Germany). Next, CD1c^+^ DCs were sorted as lineage CD11c^+^ CD1c^+^, BDCA3^+^ (CD141^+^) DCs as lineage CD11c^+^ CD141^+^, CD16^+^ DCs as lineage CD11c^+^ CD16^+^, and pDC as lineage CD11c^−^ BDCA-4^+^ by BD FACSAria III flow cytometer. As previously described (5), DC purity was assessed by double staining CD11c+/CD1c+ for CD1c^+^ DCs (>92.1%), CD11c+/CD16+ for CD16^+^ DCs (>94%), CD11c+/BDCA3+ for BDCA3^+^ DCs (>96%), and BDCA2/CD123 for pDCs (>95.3%) (Figure S1A). DCs were cultured in X-VIVO15 medium (LONZA, Switzerland) supplemented with 5% human AB serum (Haoyang, Tianjin, China), 1% penicillin streptomycin solution (Gibco, USA), 1% HEPES solution (Beyotime, China), 0.5% 2-ME (Merck, Germany), and 1% L-glutamine solution (Sigma-Aldrich, USA). DCs were stimulated with the following TLR ligands: 3 μg/mL R848 for CD1c^+^ DCs, 10 μg/mL Poly I:C for BDCA3^+^ (CD141^+^) DCs, 1 μg/mL LPS for CD16^+^ DCs and MoDC (all Sigma-Aldrich, USA), and 10 μg/mL CpG for pDCs (Novus Biologicals, USA).

### Cell Lines

The human NSCLC cell line HCC827 and H460 (ATCC, USA) were preserved by the laboratory of Cancer Center, Union Hospital, Tongji Medical College, Huazhong University of Science and Technology. HCC827 and H460 were cultured with complete RPMI-1640 medium containing 10% FBS (ScienCell, USA) and 1% penicillin streptomycin (Gibco, USA). Mutant cell line T2 (ATCC, USA), which lacks genes encoding the transporter associated with antigen processing (TAP) 1/2 (20), was cultured with 44% serum-free RPMI-1640 medium, 44% serum-free IMDM (Gibco, USA), 10% FBS (ScienCell, USA), 1% penicillin streptomycin solution (Gibco, USA), and 1% L-glutamine solution (Sigma-Aldrich, USA). Cells were washed once a month with Ficoll (Haoyang, Tianjin, China) to prevent contamination. The expression of EGFR, HLA-DR, and HLA-A2 in tumor cells were detected by flow cytometry using BD LSR II, and the expression of HLA-A2 in T2 cell lines was evaluated by BD FACSAria III flow cytometry (Figure S2).

### Peptide and Dextramer

EGFR_853−861_ (ITDF-GLAKL) (18) was synthesized by Sangon Biotech (Shanghai, China) with a purity of >95%. HLA-A^*^0201: EGFR_853−861_ Dextramer was purchased from Immudex (Copenhagen, Denmark).

### Flow Cytometry Antibodies

The following antibodies were used for flow cytometry: FITC anti-human CD3, CD16, and CD8a; PE anti-human CD4, CD8a, CD141, CD14, and CCR7; APC-eFluor780 anti-human HLA-DR; PE-Cy7 anti-human CD11c; PerCP-eFluor710 anti-human CD1c; APC anti-human CD303α; PE-Cy7 anti-human CD4; APC anti-human CD45RA and CFSE (eBioscience, USA); FITC anti-human HLA-A2 and HLA-DR; PE anti-human CD123, CD40, CD80, CD83, and CD86; APC anti-human CD137 (4-1BB); APC anti-human HLA-ABC (BD Bioscience, USA); PE anti-human EGFR; and APC anti-human CD11c (BioLegend, USA).

### Generation of Mature MoDCs

The procedure for the induction of immature MoDCs by CD14^+^ monocytes has been previously described in detail (11, 21). Briefly, PBMCs from HLA-A^*^0201 healthy volunteers were isolated with Ficoll (Haoyang, Tianjin, China). PBMCs were resuspended to 5 × 10^6^/mL with IMDM (GIBCO, USA) at 1 × 10^6^/cm^2^. Cells were seeded in a six-well cell culture plate and cultured at 37°C under 5% CO_2_. After 2 h, non-adherent cells were collected, and the six-well plate was washed with 1 × PBS buffer (Gibco, USA), incubated with IMDM for 1 h, and then washed with 1 × PBS buffer. Adherent cells were mononuclear cells cultured in complete IMDM. Complete IMDM contained 10% FBS (ScienCell), 1% penicillin streptomycin solution (Gibco, USA), 1% L-glutamine solution (Sigma-Aldrich, USA), 1,000 U/mL GM-CSM and 1,000 U/mL IL-4 (PeproTech, USA). On day 3, fresh complete IMDM was replaced, and GM-CSM and IL-4 were supplemented to 1,000 U/mL and 1,000 U/mL, respectively. On day 6, immature MoDCs were stimulated with 3 μg/mL R848, 1 μg/mL PGE2 (Sigma-Aldrich, USA), 5,000 U/mL IFN-γ, and 2.5 ng/mL TNF-α (PeproTech, USA). MoDCs were collected on day 8, and the expression levels of CD11c, CD14, CD80, CD83, CD86, CD40, HLA-DR, HLA-ABC in MoDCs were detected by BDFACS Aria III (Figure S3).

### Allogeneic Mixed Lymphocyte Reaction (MLR)

Biotin-Antibody Cocktail (including biotin-anti-human CD45RO, biotin-anti-human CD56, biotin-anti-human CD57, and biotin-anti-human CD244) and Anti-Biotin MicroBeads from PBMC non-adherent cells (95%) were used. Naïve T cells were isolated from the abovementioned T cells. Naïve CD8^+^ T cells were isolated using CD8 MicroBeads (all Miltenyi Biotec, Germany), while the remaining naïve CD4^+^ T cells were sorted as CD4^+^ CD45RA^+^ CCR7^+^ by BD FACSAria III flow cytometer and the purity was >97% (Figure S1B). Allogeneic naïve CD4^+^ T cells were labeled with 5 μM CFSE and then cocultured with CD1c^+^ DCs, BDCA3^+^ (CD141^+^) DCs, CD16^+^ DCs, pDCs, and MoDCs at a ratio of 1:10 or 1:5 with or without a TLR agonist (3 μg/mL R848 for CD1c^+^ DCs, 10 μg/mL Poly I:C for BDCA3^+^ (CD141^+^) DCs, 1 μg/mL LPS for CD16^+^ DCs and MoDCs, and 10 μg/mL CpG for pDCs.). After 6 days, T cell proliferation was assessed by flow cytometry, and cytokine secretion in the supernatant was detected by CBA (BD Bioscience, USA).

### FITC-Dextran Uptake Analysis

CD1c^+^ DCs, BDCA3^+^ (CD141^+^) DCs, pDCs, CD16^+^ DCs, and MoDCs were inoculated into U-bottom 96-well plates; 2~2.5 × 10^4^/well/200 μL medium and FITC-Dextran (Sigma-Aldrich, USA) were added to each well of the experimental group to a final concentration of 100 μg/mL. For the control group, an equal volume of 1 × PBS buffer was added per well, and cells were cultured at 4 or 37°C. After 1 h, the cells were harvested by digestion with 0.25% trypsin-0.53 mM EDTA solution (Gibco, USA). The percentage of FITC^+^ DCs was measured by BD FACSAria III flow cytometry.

### Phagocytosis Analysis of Necrotic HCC827 Cells

HCC827 cells (Sigma-Aldrich, USA) in the logarithmic growth phase were labeled with 10 μM PKH26 red fluorescent dye, and more than 97% of cells were stained (Figure S4). Staining was followed by three cycles of rapid freezing at −80°C and thawing to induce cell necrosis. Then, the cells were cocultured with CD1c^+^ DCs, BDCA3^+^ (CD141^+^) DCs, pDCs, CD16^+^ DCs, and MoDCs at 1:1 in U-bottom 96-well plates at 4 or 37°C for 12 h. Finally, we used a BD FACSAria III flow cytometer for the analysis. The swallowing ratio was measured.

### Induction, Sorting, and Amplification of EGFR_853−861_-Specific CD8^+^ T Cells

Well-grown T2 cells were seeded in six-well cell culture plates, and 10 μM EGFR_853−861_ peptide was added to each well and cultured at 37°C for 24 h. After T2 cells were pulsed with EGFR_853−861_ peptide, they were irradiated with a 40 Gy dose. Then, HLA-A^*^0201 naïve CD8^+^ T cells were mixed with irradiated T2 cells at 5:1, supplemented with 40 U/mL IL-2, and mixed. Cells were cocultured in 24-well cell culture plates with 2 mL X-VIVO 15 complete medium per well, and half of the medium was changed on day 7. T2 cells were stimulated with irradiated EGFR_853−861_ peptide again on days 11 and 18. For naïve CD8^+^ T cells, the HLA-A^*^0201: EGFR_853−861_ Dextramer^+^ CD8^+^ T cell ratio was measured on day 22, and HLA-A^*^0201: EGFR_853−861_ Dextramer^+^ CD8^+^ T cells were sorted on day 26. The cut-off point for FCM analysis was >0.005% of the CD8^+^ T cell ratio, and >10 events indicated MHC pentameric-positive CD8^+^ T cells (Figure S5). Allogeneic feeder cells were prepared as follows. Peripheral blood was collected from 3 volunteers. PBMCs were isolated, irradiated with a dose of 30 Gy, washed once, resuspended in X-VIVO 15 complete medium and mixed at 1:1:1. Feeder cells were plated in a U-bottom 96-well plate, sorted EGFR_853−861_-specific CD8^+^ T cells were seeded on feeder cells, and the ratio of T cells to feeder cells was 1:200. The system comprised 200 μL X-VIVO 15 complete medium, 3,000 U/mL IL-2, and 30 ng/mL anti-CD3, which was changed every 5 days or if necessary, and could be used for functional experiments after 3–4 weeks of stimulation.

### EGFR_853−861_-Specific CD8^+^ T Cell Response (Cross-Presentation)

#### Soluble Antigen

CD1c^+^ DCs, BDCA3^+^ (CD141^+^) DCs, pDCs, CD16^+^ DCs, and MoDCs from HLA-A^*^0201 healthy volunteers were inoculated into U-bottom 96-well plates at 2 × 10^4^/well. We added 10 μg/mL EGFR_853−861_ peptide, and cells were cultured at 37°C for 1 h. After the peptide challenge, the DC subpopulation was cocultured with 2 × 10^5^ EGFR_853−861_-specific CD8^+^ T cells with or without TLR agonists [3 μg/mL R848 for CD1c^+^ DCs, 10 μg/mL Poly I:C for BDCA3^+^ (CD141^+^) DCs, 1 μg/mL LPS for CD16^+^ DCs and MoDCs, and 10 μg/mL CpG for pDCs] for 24 h. The supernatant was collected, and the amount of secreted IFN-γ was measured by CBA.

#### Necrotic Lung Cancer Cell-Associated Antigen

CD1c^+^ DCs, BDCA3^+^ (CD141^+^) DCs, pDCs, CD16^+^ DCs, and MoDCs from HLA-A^*^0201 healthy volunteers were inoculated into U-bottom 96-well plates at 2 × 10^4^/well. Then, 2 × 10^4^ necrotic HCC827 or H460 cells was added. For some experiments, DCs were cultured with 10 μm lactacystin (Sigma-Aldrich) for 45 min before adding Ag. Each DC subpopulation was cultured at 37°C for 18 h. After ingestion of tumor cells, each DC subpopulation was cocultured with 2 × 10^5^ EGFR_853−861_-specific CD8^+^ T cells with or without TLR agonists [3 μg/mL R848 for CD1c^+^ DCs, 10 μg/mL Poly I:C for BDCA3^+^ (CD141^+^) DCs, 1 μg/mL LPS for CD16^+^ DCs and MoDCs, and 10 μg/mL CpG for pDCs] for 24 h at 37°C. The supernatant was collected, and the amount of secreted IFN-γ was measured by CBA. The cell markers CD3 and CD137 (4-1BB) were examined, and the expression of the T cell activation marker 4-1BB was detected by BD FACSAria III flow cytometry.

### Statistical Analysis

Data obtained from BD FACSAria III and BD LSR II flow cytometers were analyzed using FlowJo V10 (Tree Star, USA) and FCAP Array software (BD Bioscience, USA). Paired data were subjected to a two-tailed Wilcoxon signed rank test using GraphPad Prism 6 statistical software. Unpaired data were analyzed by a two-tailed Mann-Whitney *U*-test to determine the difference between the two groups. *P* < 0.05 was considered statistically significant. The bars and error bars in the bar graph correspond to the mean and standard deviation, respectively. The data were obtained from 3 or more independent replicate experiments.

## Results

### BDCA3^+^ (CD141^+^) DCs Are Reduced in Most Human Cancers and Correlate With Poor Prognosis

**Figure 1:**
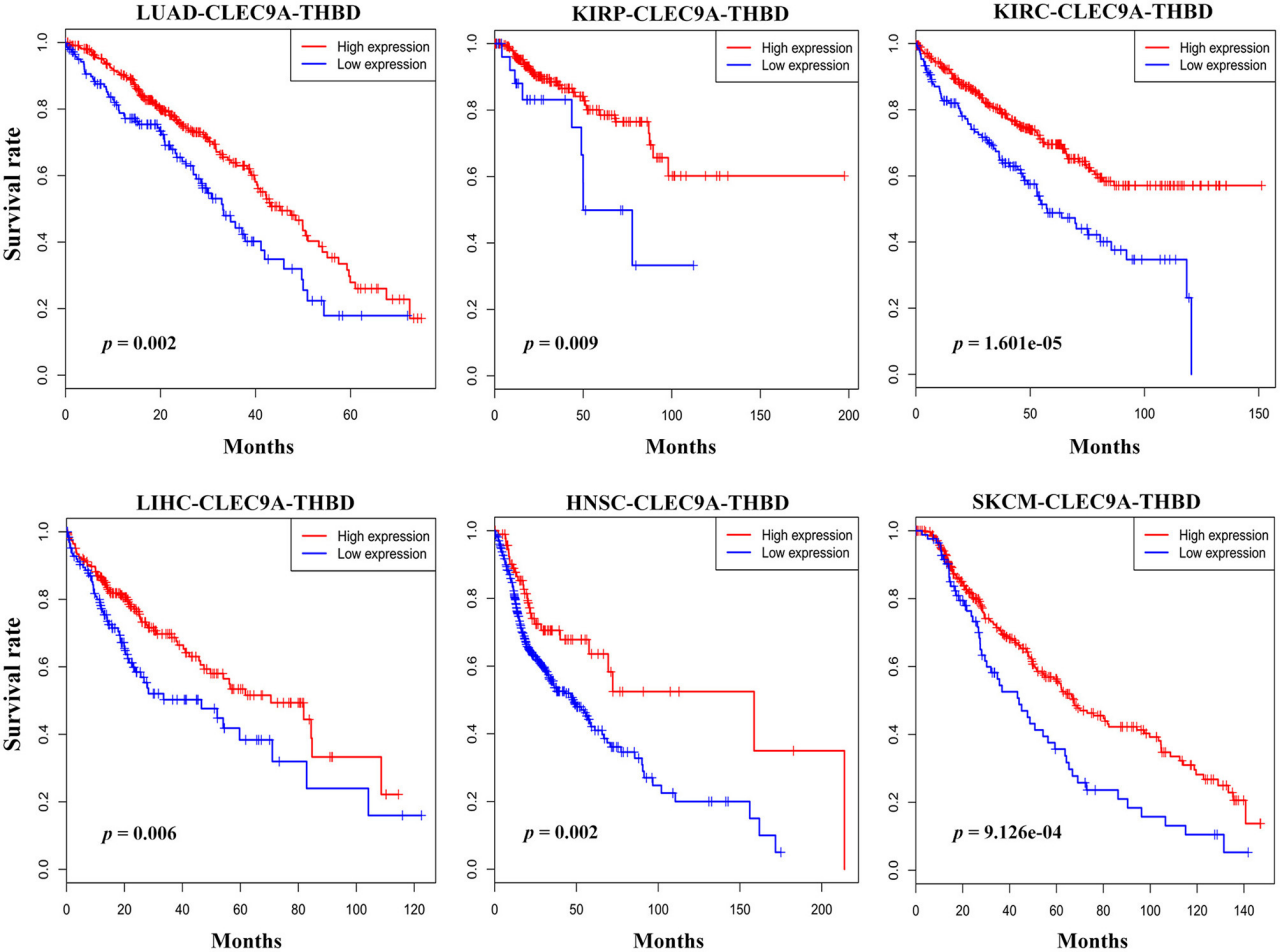
Prognostic implications of BDCA3^+^ (CD141^+^) DC signatures in several types of cancer. The geometric average of CLEC9A and THBD's expression of patients from TCGA cohorts was calculated and used as the BDCA3^+^ (CD141^+^) DC signature. Survival proportions were assessed in lung adenocarcinoma (LUAD), kidney renal papillary cell carcinoma (KIRP), kidney renal clear cell carcinoma (KIRC), liver hepatocellular carcinoma (LIHC), head and neck squamous cell carcinoma (HNSC), and skin cutaneous melanoma (SKCM) by Kaplan-Meier analysis paired with Log-rank test (Log-rank test *p* < 0.05 as a significant statistical significance). The red curve represents high BDCA3^+^ (CD141^+^) DC signature, and the blue curve represents low BDCA3^+^ (CD141^+^) DC signature. The best cutoffs are determined by X-tile software. The TCGA clinical data were downloaded from the data portal of the Genomic Data Commons (GDC), where all TCGA molecular data are also available (https://portal.gdc.cancer.gov).

Expression of CLEC9A and CD141 (THBD) is highly restricted in human BDCA3^+^ (CD141^+^) DCs. To investigated the expression of CLEC9A and THBD, and assessed their correlations with overall survival in human cancers, we searched the TCGA database. Database analyses (TCGA Research Network: http://portal.gdc.cancer.gov/) revealed that CLEC9A and CD141 were reduced in most of human cancers including lung adenocarcinoma (Figures S6, S7). The geometric average of CLEC9A and THBD's expression of patients from TCGA cohorts was calculated and used as the BDCA3^+^ (CD141^+^) DC signature. Interestingly, low BDCA3^+^ (CD141^+^) DC signature is associated with poor prognosis in lung adenocarcinoma (LUAD), kidney renal papillary cell carcinoma (KIRP), kidney renal clear cell carcinoma (KIRC), liver hepatocellular carcinoma (LIHC), head and neck squamous cell carcinoma (HNSC), and skin cutaneous melanoma (SKCM) (Figure 1). These results show that high BDCA3^+^ (CD141^+^) DC signature suggests a better prognosis.

### BDCA3^+^ (CD141^+^) DCs Induce Superior Th1 Response Compared With Other DC Subsets

The ability to induce the proliferation of allogeneic CD4^+^ T cells in an allogeneic MLR is an important feature of DCs (3). Therefore, we examined the ability of human blood CD1c^+^ DCs, BDCA3^+^ (CD141^+^) DCs, CD16^+^ DCs, CD304^+^ DCs, and MoDCs to induce CD4^+^ T cell proliferation in MLR and analyzed the effect of TLR agonists on the proliferation of T cells induced by DC subsets. To investigate the function of MoDCs in the immune responses of lung cancer, we generated immature MoDCs by CD14^+^ monocytes isolated from human PBMCs and matured them using IFN-γ, R848, PGE2, and TNF-α DCs (Figure S3). Naïve CD4^+^ T cells account for 40~60% of the total number of CD4^+^ T cells in PBMCs (Figure S8A). Almost all cells were stained with fluorescence markers after CFSE labeling (Figure S8B).

**Figure 2:**
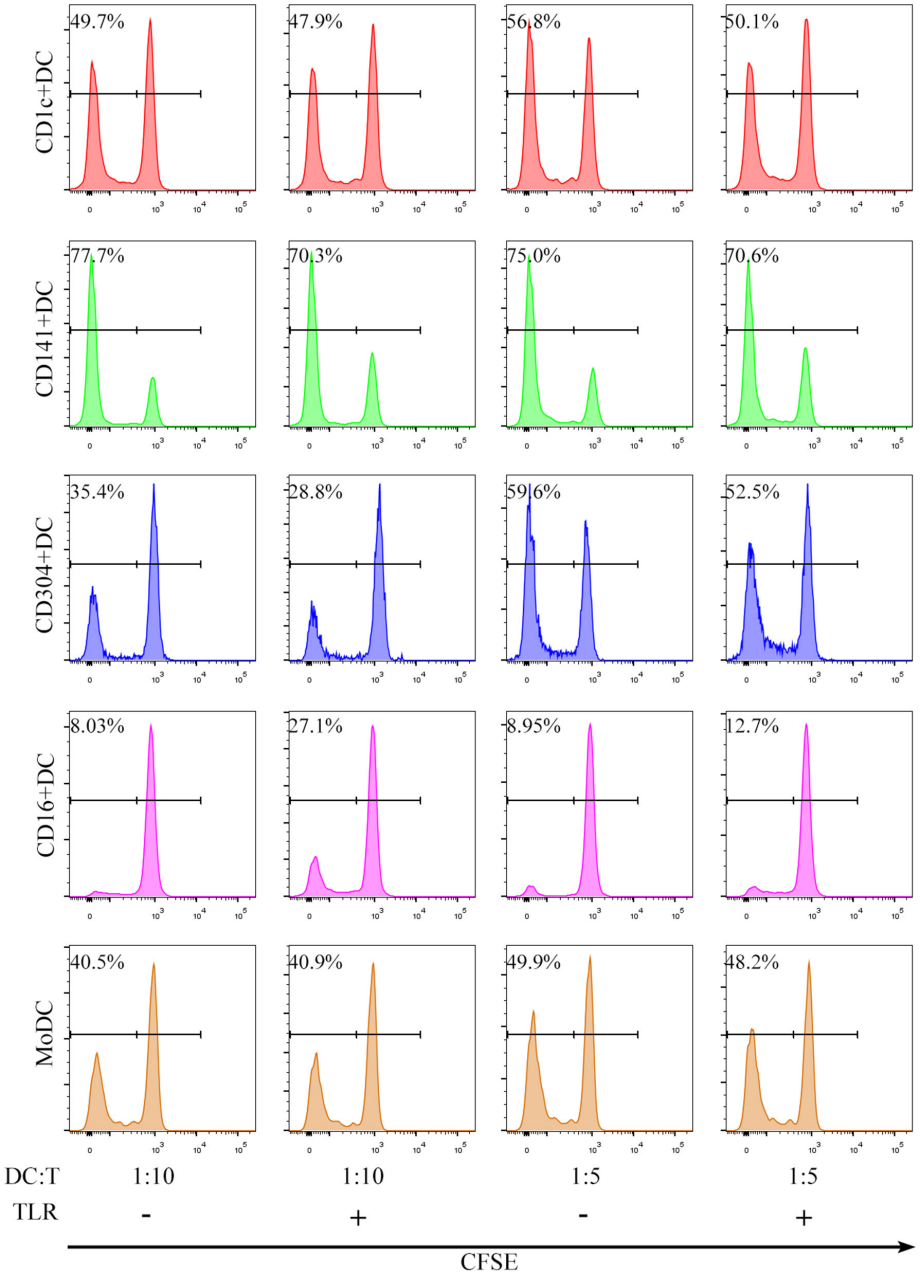
All DC subsets induce proliferation of allogeneic CD4^+^ T cells in an allogeneic MLR. CD1c^+^ DCs, BDCA3^+^ (CD141^+^) DCs, CD16^+^ DCs, CD304^+^ DCs and MoDCs from PBMCs were cultured with CFSE-labeled allogeneic naïve CD4^+^ T cells in the presence or absence of TLR agonists at a 1:5 or 1:10 ratio for 6 days. T cell proliferation was assessed by flow cytometry. One experiment out of 6 is shown.

**Figure 3:**
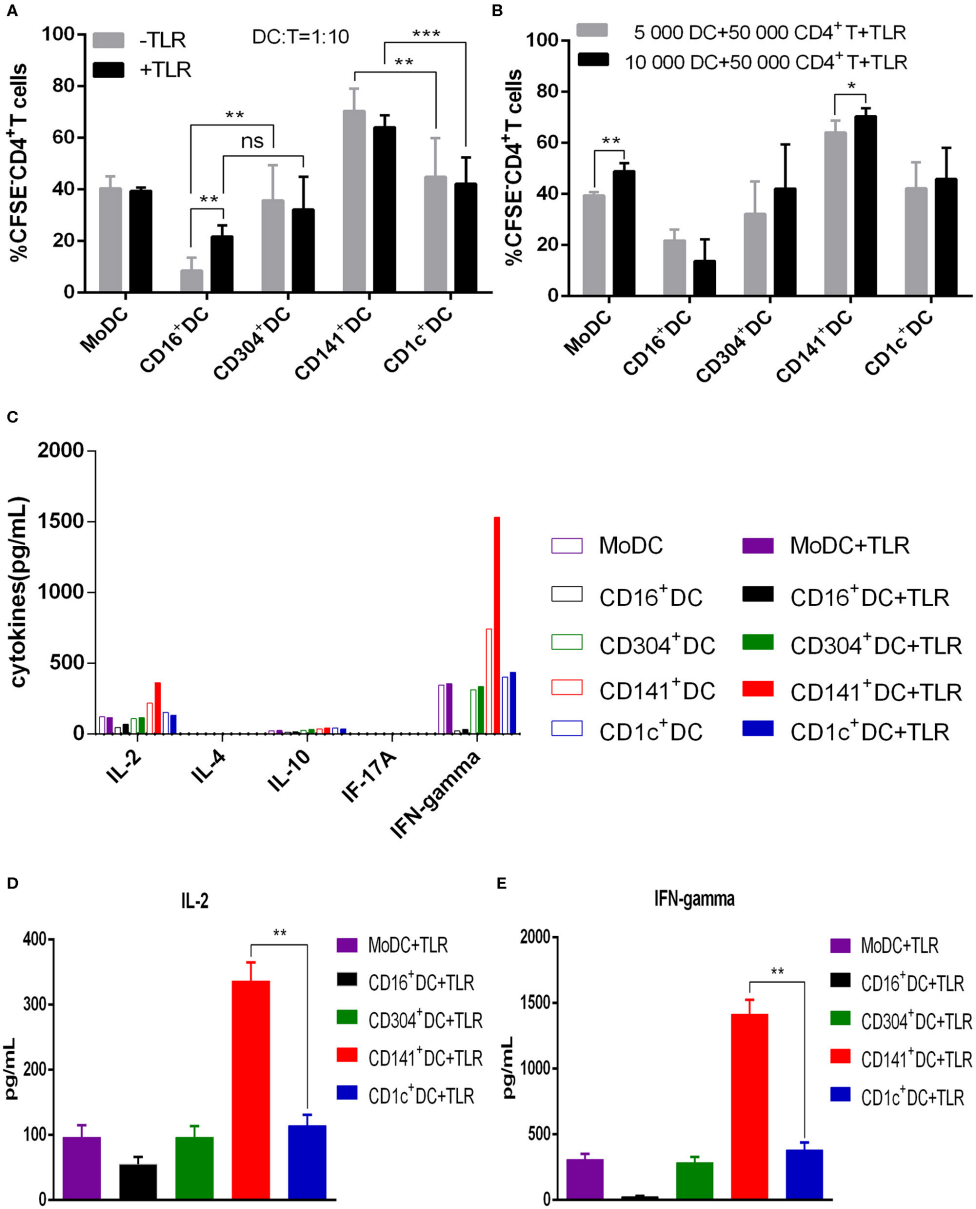
BDCA3^+^ (CD141^+^) DCs have a superior capacity to induce the proliferation of allogeneic CD4^+^ T cells. **(A)** Different DC subsets were cultured with CFSE-labeled allogeneic naïve CD4^+^ T cells in the presence or absence of TLR agonists at a 1:10 for 6 days. Then, T cell proliferation was assessed by flow cytometry. Results of five independent experiments are shown. **(B)** A total of 5,000 or 10,000 dendritic cells from different DC subsets were cultured with CFSE-labeled allogeneic naïve CD4^+^ T cells at a 1:10 or 1:5 ratio in the presence of TLR agonists. T cell proliferation was assessed by flow cytometry after 6 days. Results of six independent experiments are shown. **(C)** Dendritic cells from different DC subsets were cultured for 6 days with allogeneic naïve CD4^+^ T cells in the presence or absence of TLR agonists at a 1:10 ratio. Cytokine secretion was measured in the supernatant. One experiment out of 3 is shown. **(D,E)** Secretion of IL-2 **(D)** and IFN-γ **(E)** in the allogeneic MLR cultures after 6 days. Results of three independent experiments are shown. DCs were stimulated with or without the following TLR ligands: 3 μg/mL R848 for CD1c^+^ DCs, 10 μg/mL Poly I:C for BDCA3^+^ (CD141^+^) DCs, 1 μg/mL LPS for CD16^+^ DCs and MoDCs, and 10 μg/mL CpG for pDCs. *P < 0.05, **P < 0.01, and ***P < 0.001. NS, no significance, meaning *p* > 0.05.

All human blood DC subsets (CD1c^+^ DCs, BDCA3^+^ (CD141^+^) DCs, CD16^+^ DCs, and CD304^+^ DCs) and MoDCs stimulated the proliferation of allogenic naïve CD4^+^ T cells (Figure 2). Nevertheless, the ability of BDCA3^+^ (CD141^+^) DCs to stimulate the proliferation of CD4^+^T cells was the strongest, and the ability of CD16^+^DCs was the worst regardless of the presence of TLR agonists (Figure 3A). Interestingly, TLR agonists enhanced only the ability of CD16^+^ DCs to stimulate CD4^+^ T cell proliferation, but had no effects on other DC subsets (Figure 3A). However, when stimulated by TLR agonists, the ability of MoDCs and BDCA3^+^ (CD141^+^) DCs was enhanced by increasing the number of DCs (Figure 3B). These results suggest that BDCA3^+^ (CD141^+^) DCs were the most powerful stimulators of allogeneic CD4^+^ T cell proliferation, regardless of their activation status, and TLR agonists are dispensable in the proliferation of T cells induced by DCs.

To examine the differentiation of naïve CD4^+^ T cells stimulated by different DC subsets, we harvested the culture supernatants of DCs and T cells and detected cytokines. With exception of CD16^+^ DCs, all other DC subsets induced the secretion of the Th1 cytokine IFN-γ. All DC subsets induced different levels of IL-2, while the Th2 cytokine IL-4, regulatory T cytokine IL-10 and Th17 cytokine IL-17a were not detected (Figure 3C). These cytokines were not detected in the supernatants of DCs cultured with or without T cells (not shown in the picture). Notably, BDCA3^+^ (CD141^+^) DCs stimulate CD4^+^ T cells to secrete higher levels of IFN-γ and IL-2 compared with other DC subsets when activated by Poly I:C (Figures 3D,E). These data suggest that BDCA3^+^ (CD141^+^) DCs are strong inducers of Th1 response.

### MoDCs Take up More Macromolecules Than Do Other DC Subsets

**Figure 4:**
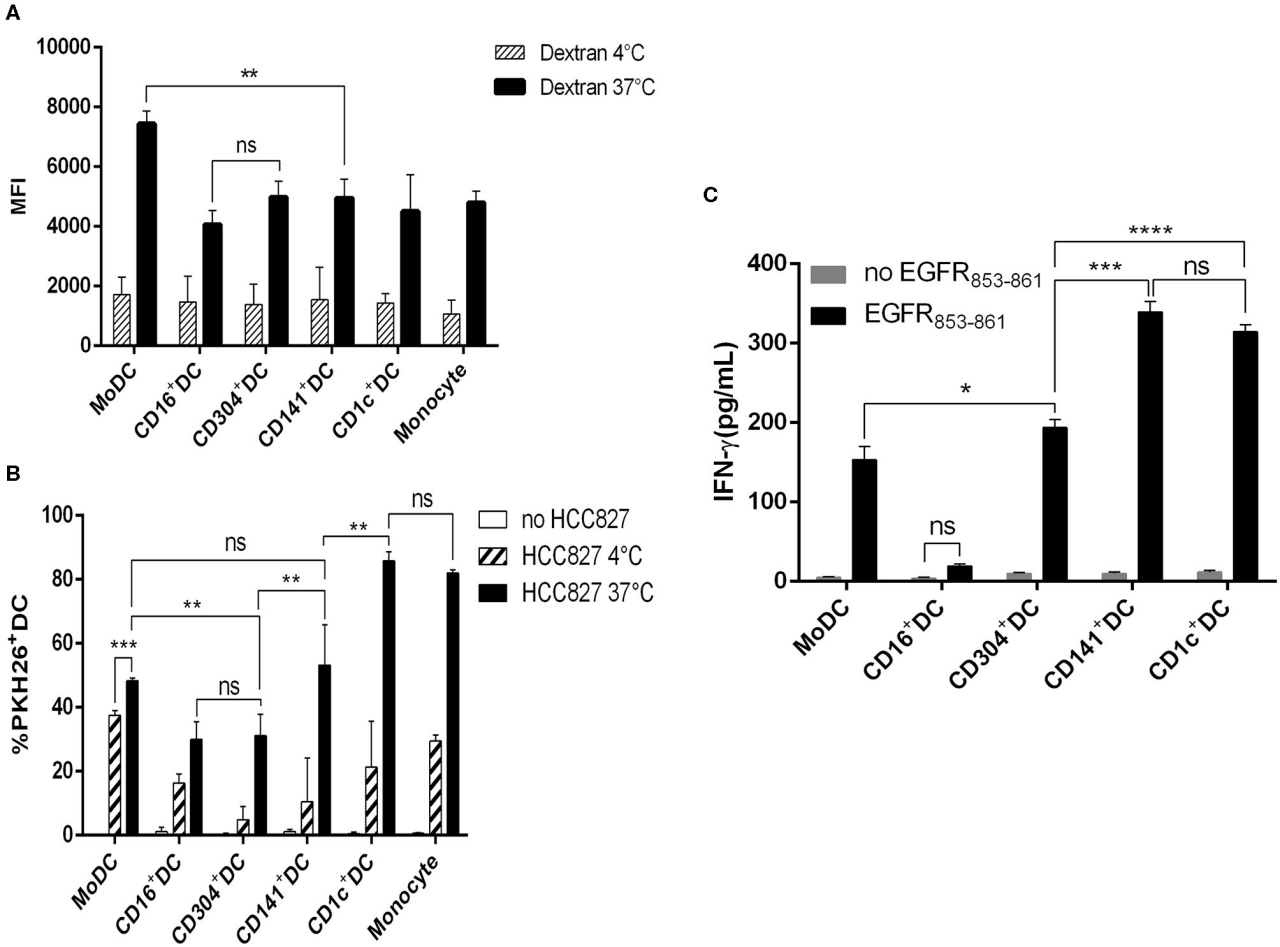
Capacity of antigen uptake and soluble antigen presentation by human DC subsets. **(A)** CD1c^+^ DCs, BDCA3^+^ (CD141^+^) DCs, CD16^+^ DCs, CD304^+^ DCs, and MoDCs were cultured for 1 h in the presence of 100 μg/mL FITC-dextran at 4 and 37°C, and then the mean fluorescence (MFI) of FITC^+^DCs was analyzed by flow cytometry. Results of three independent experiments. **(B)** Uptake of PKH-26-labeled HCC827 (the PKH26 labeled tumor cells were freeze-thawed ×3) by CD1c^+^ DCs, BDCA3^+^ (CD141^+^) DCs, CD16^+^ DCs, CD304^+^ DCs, MoDCs, and monocytes after 12 h at 4 and 37°C by flow cytometry. Results of three independent experiments. **(C)** Five DC subsets from HLA-A*0201^+^ healthy donors were loaded with HLA-A*0201-restrictive EGFR_853−861_ peptide for 2 h and used to stimulate an EGFR_853−861_-specific CTL line for 24 h. Then, the specific IFN-γ production was measured in the supernatant. Results of three independent experiments. *P < 0.05, **P < 0.01, ***P < 0.001, and ****P < 0.0001. NS, no significance, meaning *p* > 0.05.

The successful antigen presentation mediated by antigen-presenting cells includes antigen uptake, processing and presenting. Thus, the phagocytic function of DCs reflects the ability of antigen presentation to some extent. To examine the intrinsic mannose receptor-mediated endocytosis of each DC subset, we used FITC-labeled dextran particles (MW 40 kDa) as macromolecules. MoDCs were better than all the DC subsets in taking up dextran, while there was no significant difference among blood CD1c^+^ DCs, BDCA3^+^ (CD141^+^) DCs, CD16^+^ DCs, and CD304^+^ DCs (Figure 4A).

### CD1c^+^DCs Are More Efficient in Engulfing Necrotic Lung Cancer Cells

Cancer cells will invade vessels and metastasize to distant organs in the development of tumors. Cancer cells travel with blood flow and stay to form tumor metastases when flowing through a suitable habitat. Dead or dying tumor cells release cell fragments or themselves into the blood and release some chemokines that induce antigen-presenting cells to engage in phagocytosis (3). DCs acquire tumor antigens by capturing dying tumor cells or “nibbling” on live tumors (22). This acquisition will contribute to the generation of protective antitumor cytotoxic T lymphocyte responses if DCs can take up dead or dying cancer cells effectively. Therefore, we compared the ability of four blood DC subsets and induced MoDCs to phagocytose necrotic tumor cells. All DC subsets phagocytosed necrotic tumor cells, but CD1c^+^ DCs and monocytes were much more effective (Figure 4B).

### BDCA3^+^ (CD141^+^) DCs and CD1c^+^ DCs Have a Similar Capacity to Present Soluble EGFR_853−861_ Peptide

To investigate the ability of DCs to present peptide antigen to CD8+ T cells, we pulsed CD1c^+^ DCs, CD16^+^ DCs, BDCA3^+^ (CD141^+^) DCs, CD304^+^ DCs, and MoDCs from HLA-A^*^0201 donors with HLA-A^*^0201-restrictive EGFR_853−861_ peptide and used them to stimulate an EGFR_853−861_-specific CTL line. CD1c^+^ DCs, BDCA3^+^ (CD141^+^) DCs, CD304^+^ DCs, and MoDCs presented EGFR_853−861_ antigen to CD8^+^ T cells effectively. BDCA3^+^ (CD141^+^) DCs were effective and comparable to autologous CD1c^+^ DCs in their presentation of peptide antigen to CD8^+^ T cells, and other DC subsets had a weak capacity. In contrast, CD16^+^ DCs did not present peptide antigen to CD8^+^ T cells. When DCs were loaded without EGFR peptide, IFN-γ was not detected in cell culture supernatants (Figure 4C).

### BDCA3^+^ (CD141^+^) DCs Potently and Effectively Cross-Present Antigen From Necrotic Lung Cancer Cells

**Figure 5:**
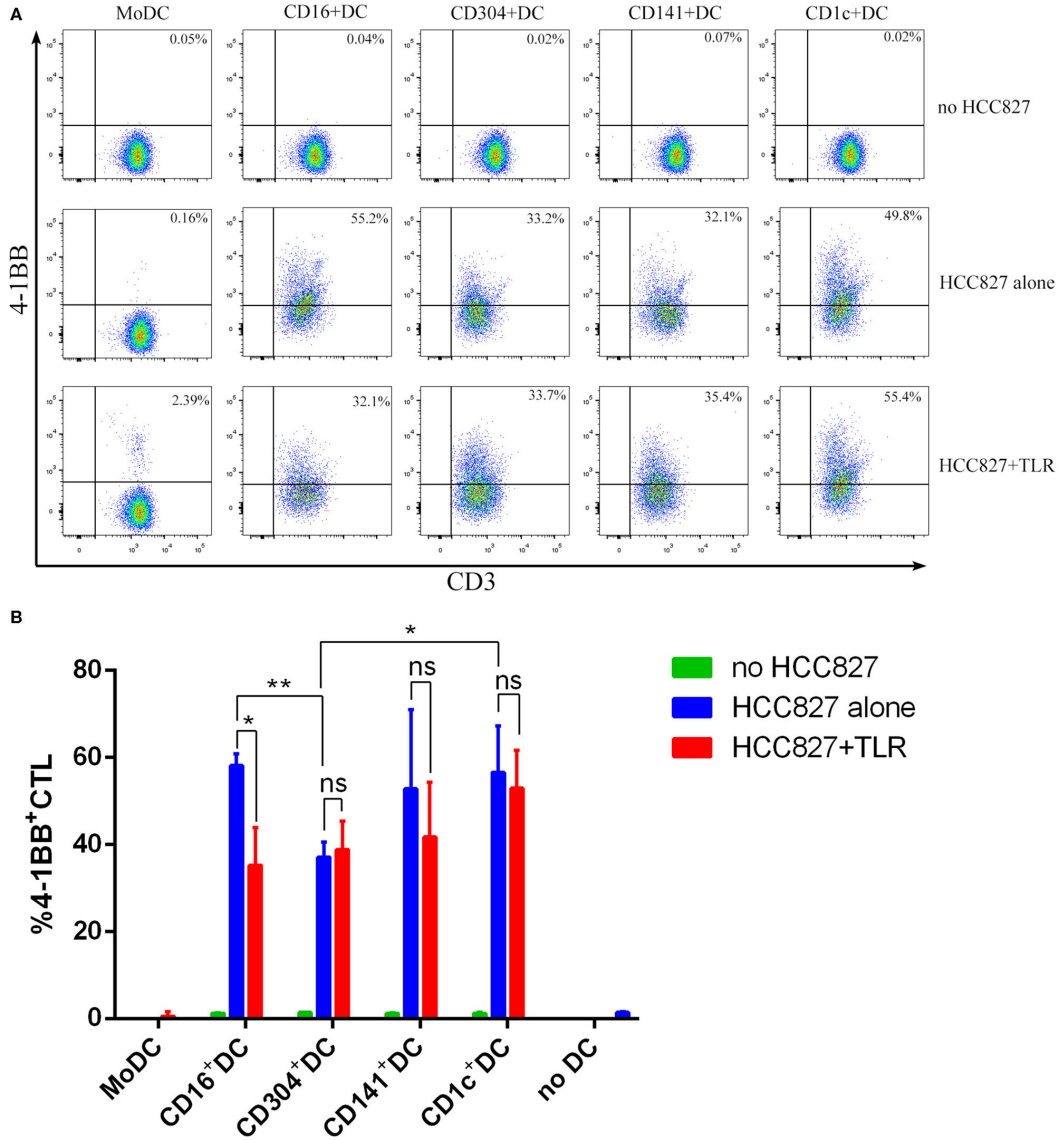
MoDCs could not stimulate EGFR_853−861_-specific CD8^+^ T cells to express 4-1BB. **(A)** Dendritic cells from HLA-A*0201 healthy volunteers were cocultured with necrotic HCC827 cells for 18 h at a 1:1 ratio and then used to activate ten-fold EGFR_853−861_-specific CD8^+^ T cells for 24 h in the presence or absence of TLR agonists [3 μg/mL R848 for CD1c^+^ DCs, 10 μg/mL Poly I:C for BDCA3^+^ (CD141^+^) DCs, 1 μg/mL LPS for CD16^+^ DCs and MoDCs, and 10 μg/mL CpG for pDCs] and analyze the expression of 4-1BB among CD8^+^ T cells. One result of three independent experiments is shown. **(B)** Results of three independent experiments are shown. *P < 0.05 and **P < 0.01. NS, no significance, meaning *p* > 0.05.

**Figure 6:**
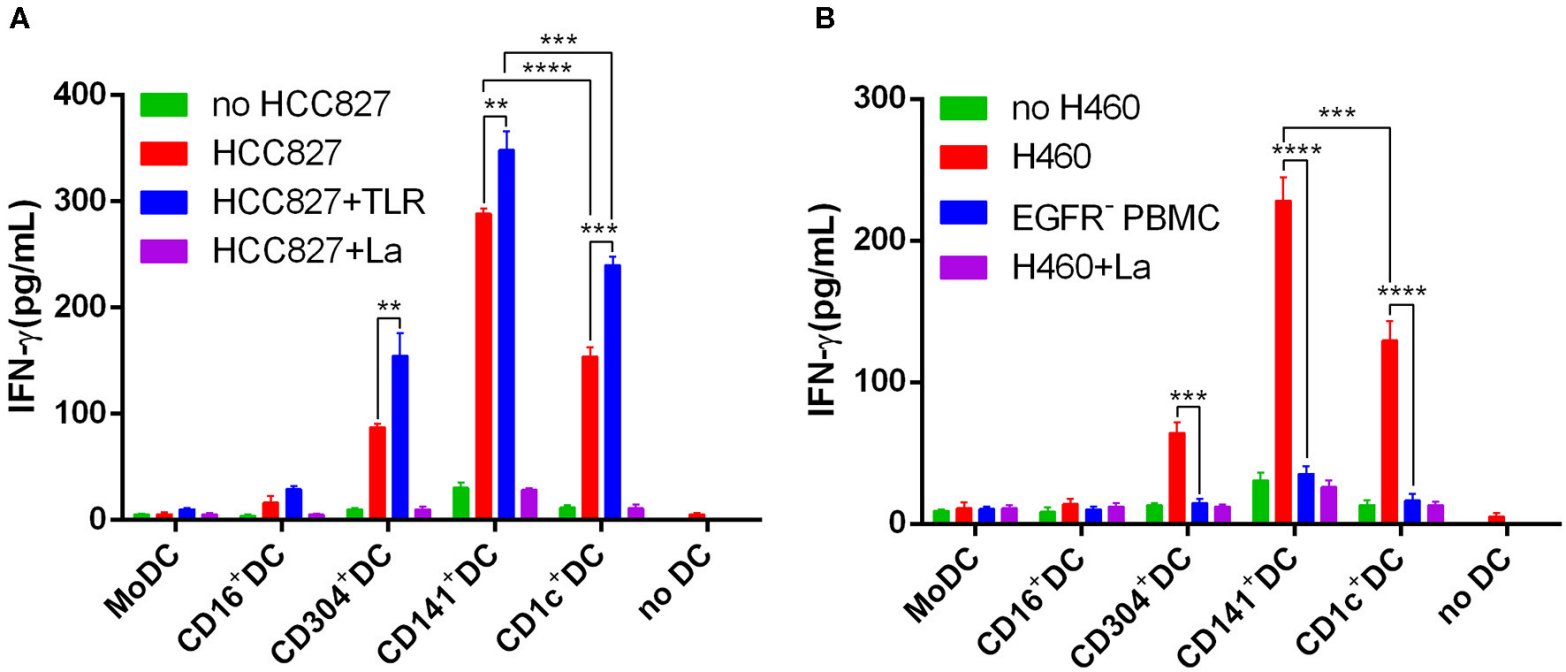
BDCA3^+^ (CD141^+^) DCs strongly and effectively cross-present antigens (EGFR_853−861_ peptide) from necrotic lung cancer cells. **(A)** Equivalent numbers of CD1c^+^ DCs, BDCA3^+^ (CD141^+^) DCs, CD16^+^ DCs, CD304^+^ DCs, and MoDCs from HLA-A*0201 healthy volunteers were incubated for 18 h with necrotic HCC827 cells at a 1:1 ratio and then used to stimulate an EGFR_853−861_-specific CD8^+^ T cell line at a 1:10 ratio in the presence or absence of TLR agonists [3 μg/mL R848 for CD1c^+^ DCs, 10 μg/mL Poly I:C for BDCA3^+^ (CD141^+^) DCs, 1 μg/mL LPS for CD16^+^ DCs and MoDCs, and 10 μg/mL CpG for pDCs]. Cross-presentation was measured as IFN-γ production by the T cell line. Cross-presentation is proteasome dependent, as it is inhibited by lactacystin (HCC827+La). Results of three independent experiments are shown. **(B)** Equivalent numbers of CD1c^+^ DCs, BDCA3^+^ (CD141^+^) DCs, CD16^+^ DCs, CD304^+^ DCs, and MoDCs from HLA-A*0201 healthy volunteers were incubated for 18 h with necrotic H460 cells at a 1:1 ratio and then used to stimulate an EGFR_853−861_-specific CD8^+^ T cell line at a 1:10 ratio in the presence or absence of TLR agonists [3 μg/mL R848 for CD1c^+^ DCs, 10 μg/mL Poly I:C for BDCA3^+^ (CD141^+^) DCs, 1 μg/mL LPS for CD16^+^ DCs and MoDCs, and 10 μg/mL CpG for pDCs]. Cross-presentation was measured as IFN-γ production by the T cell line. Cross-presentation is proteasome dependent, as it is inhibited by lactacystin (H460+La). EGFR^−^PBMCs were used as negative control. The results from 3 experiments are shown. *P < 0.05, **P < 0.01, ***P < 0.001, and ****P < 0.0001.

The human NSCLC cell line HCC827 and H460 express higher levels of EGFR than do human PBMCs (Figure S9A). In addition, HCC827 and H460 do not express HLA-A2 and HLA-DR (Figure S9B). Due to the lack of HLA-A2 and HLA-DR expression, HCC827 cells cannot present antigens to T cells. Thus, HCC827 cells do not interfere with the processing and presentation of the EGFR_853−861_ epitope from necrotic HCC827 cells by HLA-A^*^0201^+^ DCs. Therefore, the human HCC827 cell line was selected as the target antigen carrier cell to explore the ability of different DC subsets to process and present exogenous antigens. 4-1BB is expressed on activated T cells and is therefore chosen as a marker of T cell activation in this particular experiment. Among the five DC subsets, only MoDCs could not stimulate EGFR_853−861_-specific CD8^+^ T cells to express 4-1BB (Figure 5A). CD304^+^ DCs had a weaker ability to activate CD8^+^ T cells than did other DCs. However, the capacity of CD1c^+^ DCs, BDCA3^+^ (CD141^+^) DCs, and CD16^+^ DCs showed no significant differences. Interestingly, TLR agonists weakened the ability of CD16^+^ DCs to stimulate CD8^+^ T cells but had no significant effects on other DCs. CD8^+^ T cells could not be activated by any DC subset without HCC827 (Figure 5B). Although CD16^+^ DCs could stimulate CD8^+^ T cells, they did not increase the secretion of IFN-γ. BDCA3^+^ (CD141^+^) DCs, CD1c^+^ DCs, and CD304^+^ DCs could process and cross-present antigen from necrotic HCC827 cells, and BDCA3^+^ (CD141^+^) DCs were the most efficient, followed by CD1c^+^ DCs and CD304^+^ DCs. However, MoDCs neither activated CD8^+^ T cells nor cross-presented antigens from necrotic HCC827 cells. Notably, TLR ligands enhanced the ability of DC subsets to process and cross-present necrotic HCC827 cell-associated antigen. Processing of EGFR antigen by DCs was confirmed to be proteasome dependent, as it was blocked by the proteasome inhibitor lactacystin (Figure 6A). We achieved the same results by using another human NSCLC cell line H460 (Figure 6B). This demonstrated the specialized ability of BDCA3^+^ (CD141^+^) DC to cross-present antigen from necrotic cells.

## Discussion

The negative outcome of DC-based immunotherapy is closely correlated with its lack of specificity in DCs (12). However, the exact role of variant DC subsets in humans has remained a lingering question. Data from TCGA database revealed that the amount of BDCA3^+^ (CD141^+^) DCs is reduced in tumors and is positively related to the prognosis of several tumors. Thus, BDCA3^+^ (CD141^+^) DCs may have a superior ability to cross-present antigens derived from tumors and play an important role in antitumor immunity.

An exogenous antigen must be cross-presented to MHC class I molecules to initiate an adaptive anti-tumor immune response. Here, the ability of four natural DC subsets in human blood and MoDCs to cross-present antigens from necrotic NSCLC cells, as well as to directly present soluble antigen peptide, was explored. There is no difference in the ability to directly present soluble antigen peptides between BDCA3^+^ (CD141^+^) DCs and CD1c^+^ DCs, both have stronger abilities than those of the remaining DCs. Moreover, BDCA3^+^ (CD141^+^) DCs play the most important role in cross-presenting antigens from necrotic lung cancer cells.

Since TLR agonists play a fundamental role in the immunologic function of DCs (23, 24), R848, PolyI:C, LPS, and CpGs were used as adjuvants in our study. It is an important feature of DCs inducing allogeneic CD4^+^ T cell proliferation in a MLR. Thus, we evaluated this activity in different DC subsets in the present study. BDCA3^+^ (CD141^+^) DCs show the strongest ability to activate CD4^+^ T cells regardless of the presence of TLR agonists. The capacity of CD1c^+^ DCs, CD304^+^ DCs, and MoDCs to stimulate CD4^+^ T cells is similar and better than that of CD16^+^ DC. TLR agonists appear to have no effect on the activation capacity of DCs as regardless of the dosage of TLR agonists, the number of CD4^+^ T cells does not increase with that of DCs. These findings are consistent with previous reports (6).

All five DC subsets induce naïve CD4^+^ T cells to differentiate into Th1 cells, which is enhanced by TLR agonist stimulation. Our results show that among the five DC subsets, BDCA3^+^ (CD141^+^) DCs mediate the strongest Th1 response, which was consistent with the research of Jongbloed et al. (6). However, another study found that human BDCA3^+^ (CD141^+^) DCs can induce the differentiation of naïve CD4^+^ T cells into Th2 cells (25). In this study, after being cocultured with BDCA3^+^ (CD141^+^) DCs stimulated by live attenuated influenza vaccine, naïve CD4^+^ T cells differentiated into both Th1 and Th2 cells. Since the function of DCs varies according to different stimulants, BDCA3^+^ (CD141^+^) DCs might have significant plasticity in inducing an adaptive immune response.

To some extent, DC phagocytosis determines its antigen recognition processing. In our study, MoDCs are better at endocytosing macromolecules, such as FITC-labeled dextran, than the remaining four DC subsets. Melanoma, testicular cancer or colorectal cancer cells that were exposed to cisplatin or oxaliplatin could enhance the phagocytosis of CD16^+^ DCs, CD1c^+^ DCs, and pDCs and encourage them to gain a mature phenotype (26). Among the peripheral blood origin DC subsets, pDCs have a weak ability to phagocytize cell debris (5). However, there was no remarkable difference in the capacity of engulfing necrotic fibroblast fragments transfected by human cytomegalovirus between BDCA3^+^ (CD141^+^) DCs and CD1c^+^ DCs (6). These studies show the inconsistencies in the ability of DC subsets to phagocytose necrotic cell fragments, while which type of DC exhibits the best phagocytosis abilities remains unclear. In this study, we investigated in detail the phagocytosis ability of different DC subsets and compared it with that of monocytes. In contrast to previous studies, we found that the capacity of CD1c^+^ DCs to phagocytose necrotic lung cancer cells is similar to that of monocytes but stronger than that of other DCs. CD16^+^ DCs and pDCs have the worst phagocytosis capacity. This result may be due to different functional remodeling processes of DC subsets against various antigens.

The efficacy of antigen presentation is determined by several key steps, including antigen uptake, processing, loading onto MHC I molecules, presenting, and cross-activating CD8^+^ T cells. Synthetic antigen epitope peptides could be directly presented to antigen-specific CD8^+^ T cells by DCs without processing. Many studies have confirmed that soluble epitopes can be presented directly to antigen-specific CD8^+^ T cells by BDCA3^+^ (CD141^+^) DCs, CD1c^+^ DCs, and pDCs (5, 6, 24). The presentation capacity of BDCA3^+^ (CD141^+^) DCs is comparable to that of CD1c^+^ DCs (6). Nonetheless, these studies mainly focused on DC functions in human cytomegalic virus diseases and melanoma, which show strong immunogenicity, while those in lung cancer and other solid tumors that have weaker immunogenicity remain to be explored. For this important reason, we carried out the present study. In addition to CD16^+^ DCs, BDCA3^+^ (CD141^+^) DCs, CD1c^+^ DCs, pDCs, and MoDCs can directly present soluble epitope peptide to antigen-specific CD8^+^ T cells. BDCA3^+^ (CD141^+^) DCs have the same capacity as CD1c^+^ DCs, which is stronger than that of other DCs.

In mice, it was first confirmed that DC subsets exert unique effects on the outcome of the immune responses. CD8a^−^ DCs and CD8a^+^ DCs are two classical DCs that reside in the lymphoid organs of mice. The former induces antigen-specific CD4^+^ T cell response preferentially, while the latter induces antigen-specific CD8^+^ T cell response (27). A large number of studies have shown that CD8a^+^ DCs play a vital role in inducing protective CD8^+^ cytotoxic T cell response, which are essential for radical curing of malignant tumors, viruses, and other pathogen infections (27, 28). CD8α^+^ DCs in mice produce IL-12p70 and mediate Th1-type cytokines such as IL-2 and IFN-γ (29–32). More importantly, CD8α^+^ DCs in mice have a unique antigen cross-presenting capability, especially after taking up dead or dying cells (33–36). Since there are no CD8α^+^ DCs in humans, the aforementioned results of mouse DCs cannot be directly translated into clinical applications. Nonetheless, human BDCA3^+^ (CD141^+^) DCs share many similar phenotypes with mouse CD8^+^ DCs as both DCs express TLR3, nectin-like protein 2 (Necl2), and CLEC9A (6). Therefore, some researchers believe that BDCA3^+^ (CD141^+^) DCs in humans and CD8^+^ DCs in mice might have similar functions (6). Nevertheless, notably, there are remarkable differences between DCs from humans and mice as mouse mDCs express TLR9 and secrete IFN-α (30, 37), and human pDCs have similar functions (38). Nizzoli et al. found that under the stimulation of TLR agonists, only CD1c^+^ DCs produce IL-12p70 (24). Jongbloed et al. reported that after stimulation, BDCA3^+^ (CD141^+^) DCs secrete a small amount of IL-12p70 (6). Although CLEC9A, which participates in taking up and cross-presenting antigens (39), is expressed only in BDCA3^+^ (CD141^+^) DCs among all human DCs, which subsets are more capable of cross-presenting antigens remains controversial (5, 6, 26). In our study, BDCA3^+^ (CD141^+^) DCs, CD1c^+^ DCs, and pDCs could cross-present antigens from necrotic lung cancer debris, and TLR agonists could further enhance this process. According to our research, BDCA3^+^ (CD141^+^) DCs exhibit the strongest cross-presentation ability, followed by CD1c^+^ DCs, while CD16^+^ DCs and MoDCs cannot present antigens derived from necrotic lung cancer cells. Although CD16^+^ DCs can activate specific CTLs, they cannot stimulate the secretion of IFN-γ. Recently, an analysis of the whole genome expression spectrum suggested that CD16^+^ DCs might not be real DCs but rather a unique monocyte subset with DC characteristics (40). Our results are consistent with the above study.

DC-based immunotherapy for lung cancer has become a novel research focus in recent years (41–46). Exploring the immune responses of different DC subsets will help optimize the DC vaccine for cancer treatment. Our results suggested that BDCA3^+^ (CD141^+^) DCs may play a crucial role in antitumor immune responses, these cells would be the most suitable for creating DC cancer vaccines. Regulating DCs through a combination of DC-specific antibodies with specific antigens *in vivo* may be a feasible idea, as the separation of BDCA3^+^ (CD141^+^) DCs *in vitro* is currently impractical.

## Conclusions

In conclusion, our study demonstrates that human BDCA3^+^ (CD141^+^) DCs have the strongest ability to induce Th1 cell differentiation among five DCs subsets. More importantly, human BDCA3^+^ (CD141^+^) DCs are better at presenting soluble antigen peptides to antigen-specific CTLs as well as cross-presenting antigens from necrotic lung cancer cells than the other DC subsets. Our results provide a novel perspective for the application of DC-based anticancer vaccines.

## Data Availability Statement

The datasets generated for this study are available on request to the corresponding author.

## Ethics Statement

The studies involving human participants were reviewed and approved by the Ethics Committee of Tongji Medical College, Huazhong University of Science and Technology. The patients/participants provided their written informed consent to participate in this study. Written informed consent was obtained from the individual(s) for the publication of any potentially identifiable images or data included in this article.

## Author Contributions

LL conceived and designed the experiments. FG, YL, YH, JL, YZ, and QY performed the experiments. FG and YW analyzed and interpreted the data. FG, LM, and KZ were in charge of manuscript writing. LL supervised the experimental work and revised the manuscript. All authors read and approved the manuscript.

## Conflict of Interest

The authors declare that the research was conducted in the absence of any commercial or financial relationships that could be construed as a potential conflict of interest.
