# Supplementary material for: The Superior Ability of Human BDCA3+ (CD141+) Dendritic Cells (DCs) to Cross-Present Antigens Derived From Necrotic Lung Cancer Cells
Source: Front Immunol. 2020 Jun 19;11:1267. doi: 10.3389/fimmu.2020.01267 (PMC7325999; doi:10.3389/fimmu.2020.01267)
Supplement: Supplementary file 1 [file Data_Sheet_1.PDF]

Supplementary Figures

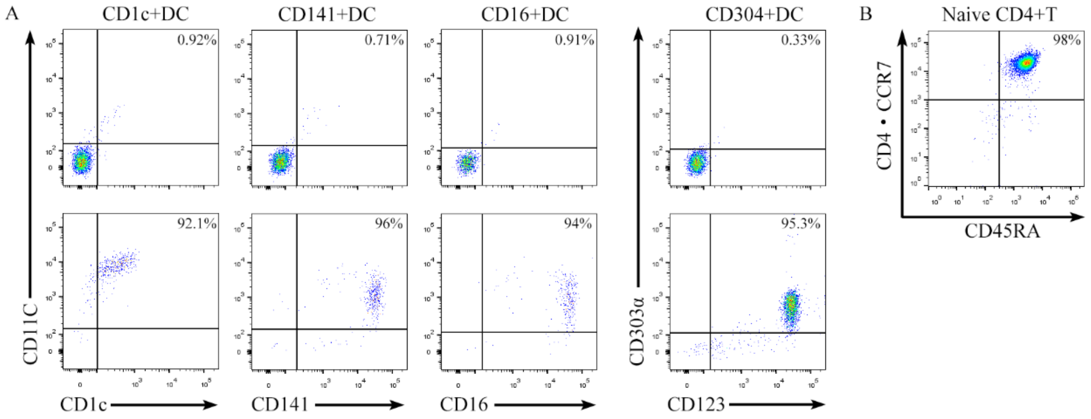

Fig S1. The purity of different DC subsets and naïve CD4$^+$T cells. A. DC purity was assessed by double staining for CD11c$^+$/CD1c$^+$ for CD1c$^+$ DCs, CD11c$^+$/CD16$^+$ for CD16$^+$ DCs, CD11c$^+$/BDCA3$^+$ for BDCA3$^+$ (CD141$^+$) DCs, and BDCA2/CD123 for pDCs. Staining was analyzed by flow cytometry. The control group was stained without any flow cytometry antibodies. B. The purity of naïve CD4$^+$T cells was assessed by staining for CD4, CD45RA and CCR7.

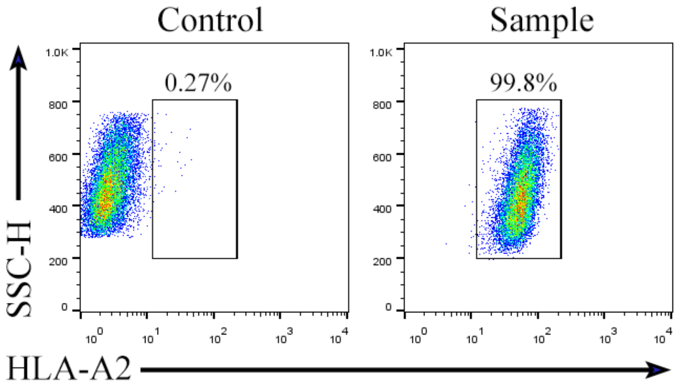

Fig S2. T2 cells express high levels of HLA-A2. T2 cells were obtained and stained with anti-HLA-A2 and analyzed by flow cytometry.

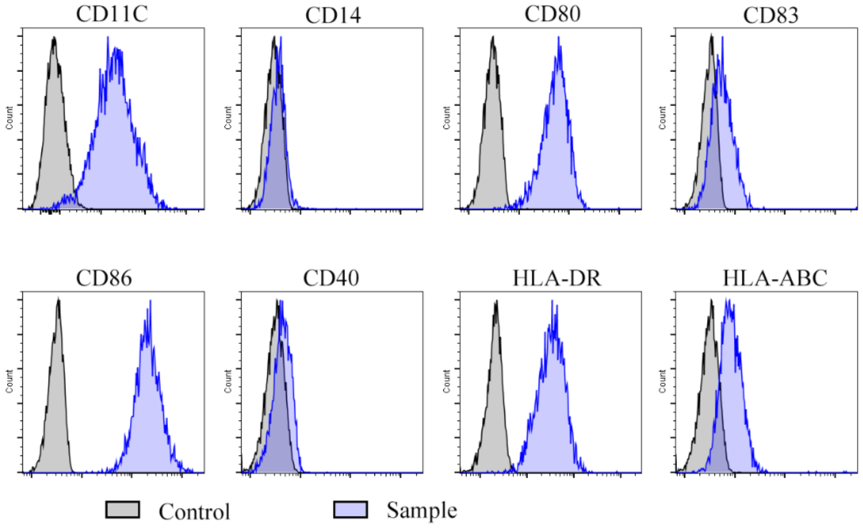

Fig S3. Characteristics of mature MoDCs. Human CD14$^+$ monocytes isolated from

PBMCs by plastic adherence were cultured for 6 days in the presence of GM-CSF and IL-4 and then activated with a maturation cocktail comprising IFN-γ, R848, PGE2 and TNF-α. Cells were harvested on day 8, and the expression of CD11C, CD14, CD80, CD83, CD86, CD40, HLA-DR and HLA-ABC was examined by flow cytometry.

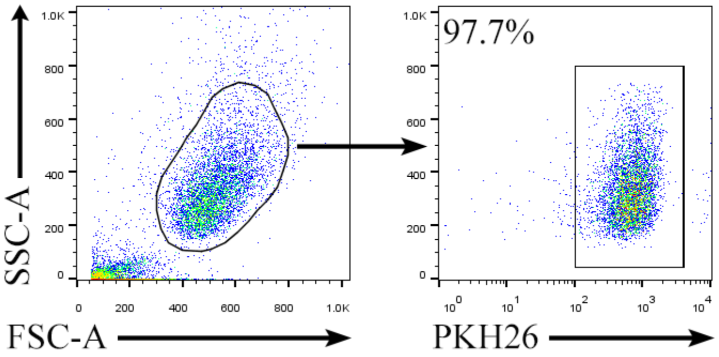

Fig S4. HCC827 cells stained with PKH26 red fluorescence. HCC827 cells were obtained, stained with PKH26 and analyzed by flow cytometry.

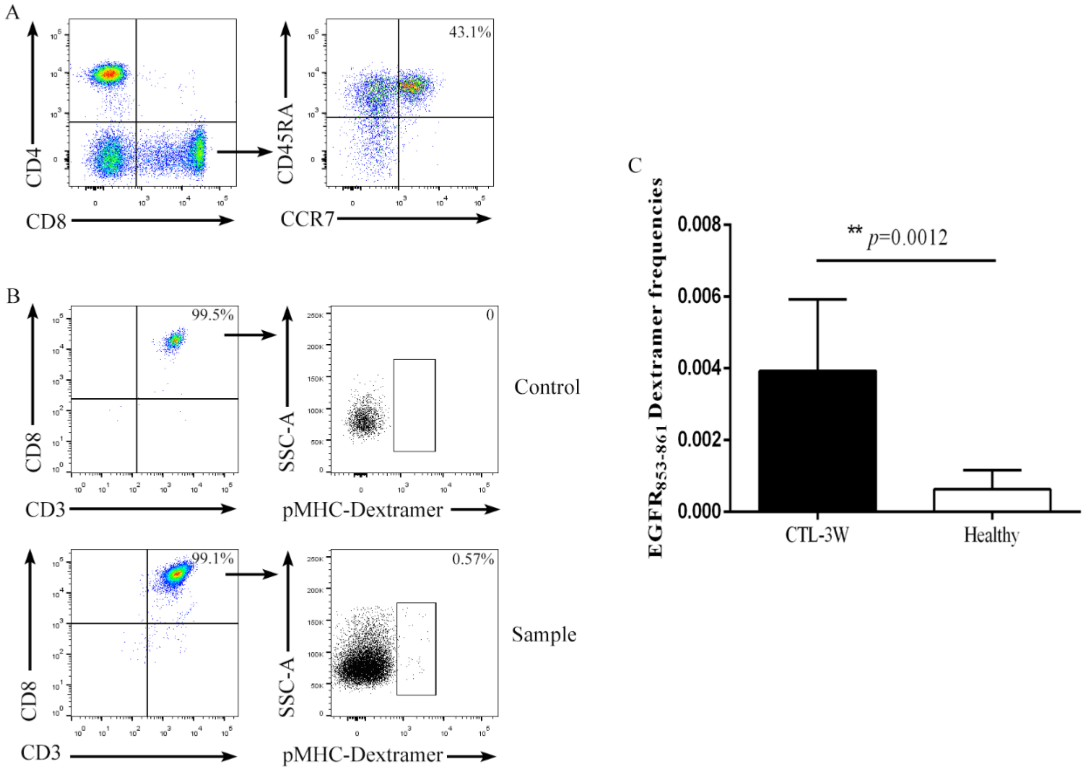

Fig S5. Induction of EGFR$_{853\text{-}861}$-specific CD8$^+$ T cells. A. PBMCs from HLA-A*0201$^+$ healthy donors were stained with anti-CD4, CD8, CD45RA and CCR7 and analyzed by flow cytometry. Naïve CD8$^+$ T cells were gated as CD4$^-$CD8$^+$CD45RA$^+$CCR7$^+$. B. After stimulation by irradiated T2 cells loaded with EGFR$_{853\text{-}861}$ for 3 weeks, CD8$^+$ T cells were stained with anti-CD3, CD8 and HLA-A*0201: EGFR$_{853\text{-}861}$ Dextramer and analyzed by flow cytometry. One representative experiment out of 5 is shown. C. The percentage of EGFR$_{853\text{-}861}$-specific CD8$^+$ T cells among CD3$^+$CD8$^+$ T cells after induction for 3 weeks. The results from 5 experiments are shown.

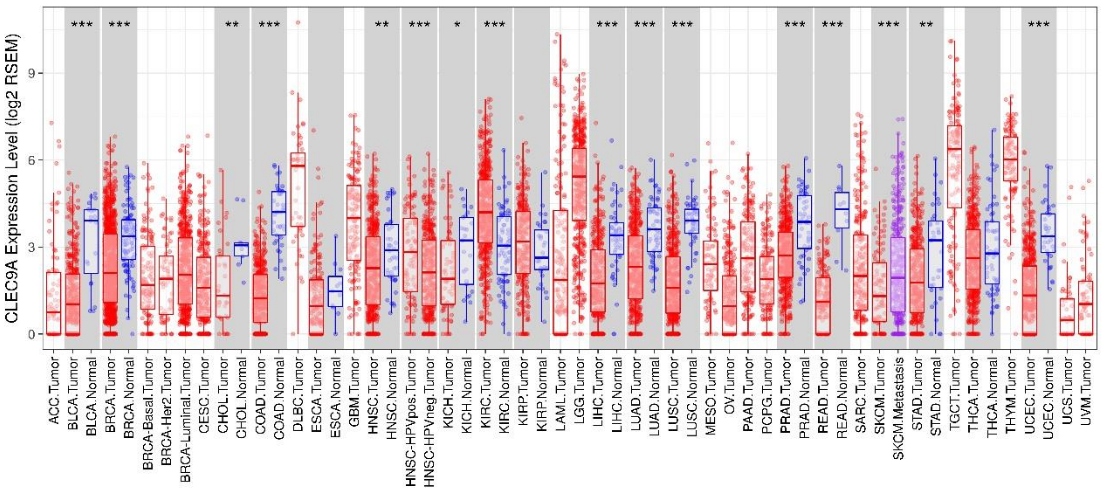

Fig S6. The expression of CLEC9A is reduced in most human cancers. Expression of CLEC9A between tumor and adjacent normal tissues across all TCGA tumors. Distributions of gene expression levels are displayed using box plots, with statistical significance of differential expression evaluated using Wilcoxon test. CLEC9A genes that are up- or down- regulated in the tumors compared to normal tissues for each cancer type was displayed in gray columns when normal data are available. (P-value Significant Codes: *** < 0.001 ≤ ** < 0.01 ≤ * < 0.05).

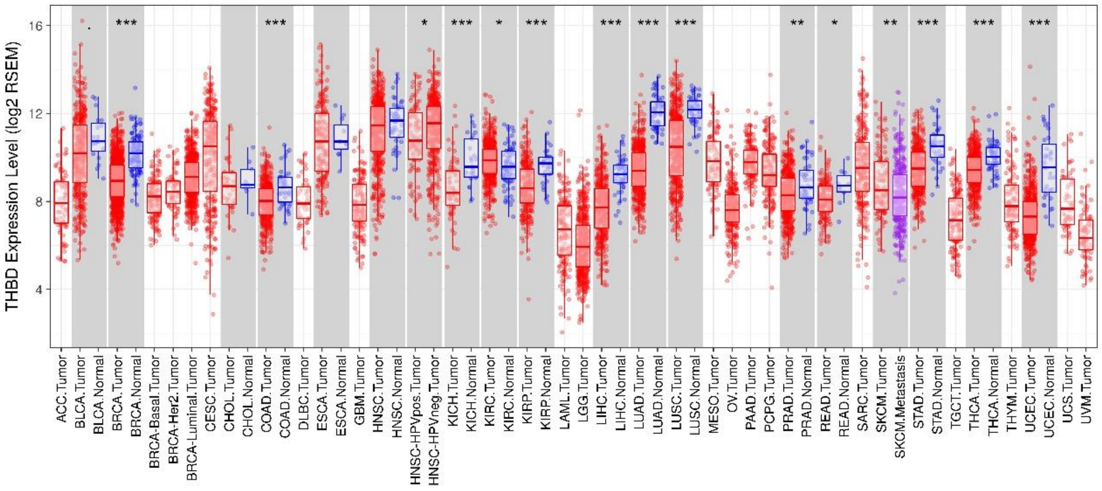

Fig S7. The expression of CD141 (THBD) is reduced in most human cancers. Expression of CD141 (THBD) between tumor and adjacent normal tissues across all TCGA tumors. Distributions of gene expression levels are displayed using box plots, with statistical significance of differential expression evaluated using Wilcoxon test. CD141 (THBD) genes that are up- or down- regulated in the tumors compared to normal tissues for each cancer type was displayed in gray columns when normal data are available. (P-value Significant Codes: *** < 0.001 ≤ ** < 0.01 ≤ * < 0.05).

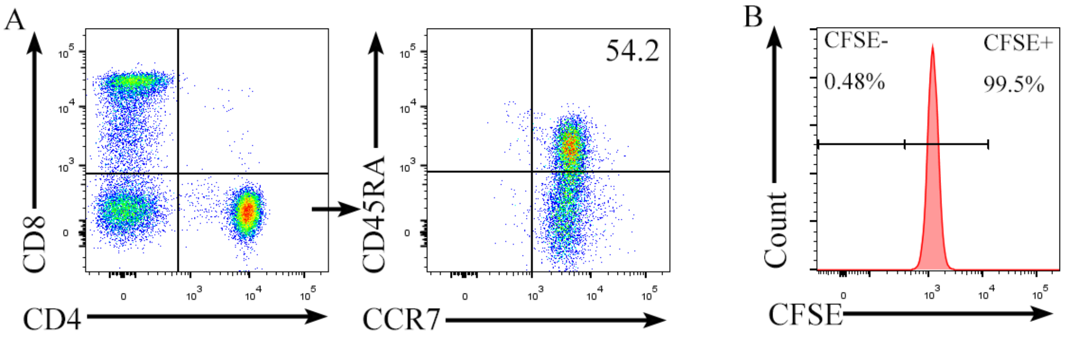

Fig S8. The percentage of naïve $CD4^+$T cells in PBMCs. A. PBMCs were stained with anti-CD4, CD8, CD45RA and CCR7 and analyzed by flow cytometry. Naïve $CD4^+$ T cells were gated as $CD4^+CD8^-CD45RA^+CCR7^+$. B. Naïve $CD4^+$ T cells isolated from PBMCs using MACS were labeled with 5 μM CFSE and then assembled by flow cytometry. One representative experiment out of 3 is shown.

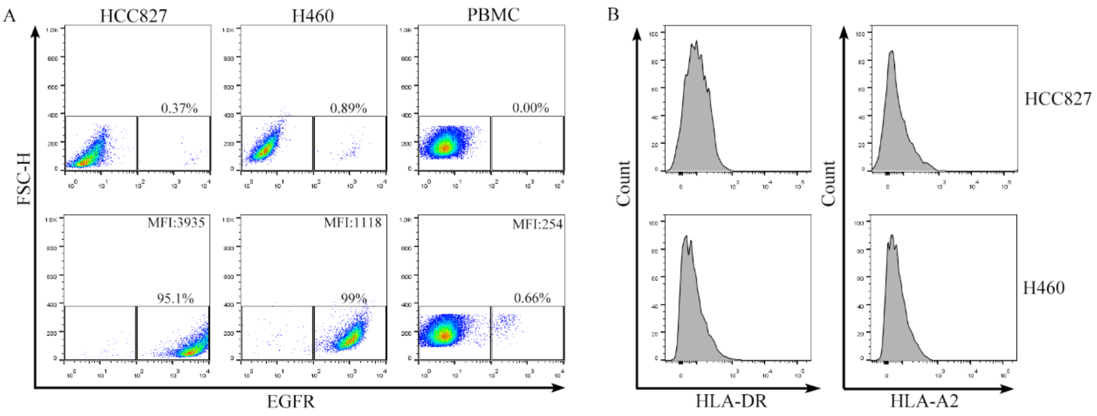

Fig S9. Characteristics of the human HCC827 and H460 cell line. A. Human HCC827 cells, H460 and PBMCs were stained with anti-EGFR and analyzed by flow cytometry. B. HCC827 and H460 cells were stained with anti-HLA-DR and HLA-A2 and analyzed by flow cytometry. One representative experiment out of 3 is shown.
